# Caffeic Acid Phenethyl Ester (CAPE) Improves Boar Sperm Quality and Antioxidant Capacity in Liquid Preservation (17°C) Linked to AMPK Activity Maintenance

**DOI:** 10.3389/fvets.2022.904886

**Published:** 2022-06-09

**Authors:** Qun Lan, Li'e Xue, Jiacheng Cao, Yingyu Xie, Tianfang Xiao, Shaoming Fang

**Affiliations:** College of Animal Science (College of Bee Science), Fujian Agriculture and Forestry University, Fuzhou, China

**Keywords:** boar sperm, liquid preservation, CAPE, AMPK, sperm quality, antioxidant capacity

## Abstract

Liquid preservation of boar sperm is crucial for artificial insemination application in pig production. However, time-dependent oxidative damage to sperm is one of the major challenges during the liquid preservation period. Caffeic acid phenethyl ester (CAPE) possesses excellent antioxidant properties and has potential therapeutic use in reproductive organ injury linked to oxidative stress. Adenosine monophosphate (AMP)-activated protein kinase (AMPK) involves in modulating the cellular redox state and exerts a beneficial effect on sperm preservation. In the present study, we firstly assessed different concentrations of CAPE that affect sperm quality during liquid storage to determine the appropriate addition. To further investigate whether CAPE exerts protective effects on boar sperm through modulation of AMPK activity, sperm quality parameters, antioxidant capacity, and marker protein expressions were evaluated under co-incubation with H_2_O_2_. The results showed that sperm treated with 210 μmol/L CAPE exhibited the highest motion parameters (total motility and progressive motility) and best functional integrity (mitochondrial activity, plasma membrane integrity, and acrosomal integrity). Even in the presence of H_2_O_2_, the addition of 210 μmol/L CAPE not only significantly improved sperm quality parameters, but also elevated CAT, SOD, and GSH-Px activities to enhance sperm antioxidant capacity. In addition, we found that CAPE could affect the protein activities of AMPK, phospho-AMPK α (p-AMPK), SOD, and Caspase-3 regardless of whether H_2_O_2_ is present or not. Our findings suggested that CAPE has potential application in liquid preservation of boar sperm and preliminary indicated that CAPE-induced improvement of sperm quality and antioxidant capacity should be mediated through conservation of AMPK activity. Further studies are required to illustrate the specific mechanism by which CAPE attenuates oxidative stress-mediated damages dependent on AMPK activity.

## Graphical Abstract

The potential protective mechanism of CAPE on boar sperm. CAPE induces AMPK phosphorylation resulting in elevating the protein expression of SOD1 to eliminate ROS as well as inhibiting the protein expression of Caspase-3. Consequently, both the liquid preservation time and boar sperm quality are improved. 
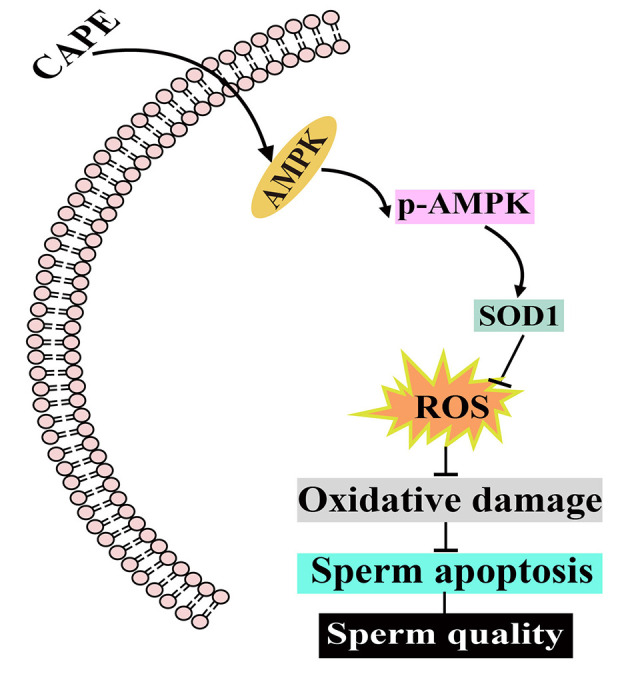

## Introduction

In pig production, artificial insemination (AI) is applied for breeding over 90% of the sows (1). Hence, the effective preservation of boar sperm is vital for its utilization in artificial insemination. The liquid preservation of boar sperm is a common sperm preservation technology that is efficient in maintaining therm motility and fertilization capacity for days. Nonetheless, a major problem in the long-time liquid preservation of boar semen is free radicals and reactive oxygen species (ROS) accumulation which results in oxidative damage to sperm (2). Thus, extensive attention has been devoted to finding appropriate antioxidants to protect boar sperm from oxidative damage during liquid storage (3–5).

Caffeic acid phenethyl ester (CAPE) is a critical bioactive polyphenolic compound of propolis (6). Due to its broad spectrum of biological properties including antioxidant, anti-inflammatory, antibacterial, and immunomodulatory effects, CAPE has received great attention in the last few decades. More recently, CAPE is regarded as a promising therapeutic agent for reproductive functions and oxidative stress-based pathologies of gonads (7). Celik et al. reported that CAPE ameliorated reperfusion injury in the rat ovary by decreasing malondialdehyde (MDA) and xanthine oxidase (XO) activities and by increasing reduced glutathione (GSH) levels (8). Abdallah et al. revealed that owing to the antioxidant properties of CAPE, it reduced oxidative damage of rat testes induced by lambda-cyhalothrin (LC) and improved fertility (9). Ayla et al. suggested that preincubation of human spermatozoa with CAPE resulted in a significant reduction of MDA level, which provided protection against oxidative stress-mediated sperm dysfunction and DNA damage (10). On the other hand, adenosine monophosphate (AMP)-activated protein kinase (AMPK) is a key kinase that participated in regulating the cellular redox state by altering the metabolic pathway under stressful conditions, which has a beneficial role in sperm preservation (11). Nashtaei et al. indicated that the increased AMPK activity enhanced the mitochondrial functions and was important for protection against cryopreservation-induced oxidative stress of human spermatozoa (12). Zhu et al. in both boar and goat spermatozoa studies found that the activation of AMPK allows the reduction of ROS production while reinforcing the sperm antioxidative capacity such as increasing GSH level and activities of glutathione peroxidase (GSH-Px), superoxide dismutase (SOD), and catalase (CAT) (13, 14). Therefore, modulation of AMPK activity should be a good way in sperm preservation strategy. More importantly, CAPE is capable of activating AMPK *in vivo* and *in vitro* has been reported in earlier researches (15, 16).

In this study, different concentrations of CAPE that affect sperm quality during liquid storage at 17 °C were evaluated to determine the optimal addition. Additionally, in the presence or absence of H_2_O_2_, sperm quality parameters, antioxidant capacity, and indicator protein expressions were analyzed to investigate whether CAPE exerts protective roles in boar sperm via regulating AMPK activity. Our findings could offer basic knowledge for CAPE utilization in the liquid preservation of boar sperm and provide the first evidence for CAPE protects boar sperm from oxidative damage by conserving AMPK activity.

## Materials and Methods

### Animal and Chemical

The semen samples were collected from eight healthy and sexually mature (8–10 months old) Landrace boars in the commercial breeding pig farm (YongCheng Agricultural&Animal Husbandry Sci-Tech Co., Ltd, Fujian, China). Verbal consent for the sample collection was obtained from the owner of the experimental pigs. Except CAPE was purchased from Shanghai Macklin Biochemical Co., Ltd. (Shanghai, China), the other chemicals and reagents used in the present study were purchased from Sigma-Aldrich Co., Ltd. (Shanghai, China).

### Semen Collection and Extender Preparation

Traditional gloved hand methodology was used to collect boar semen ejaculates. The procedures were described in our previous study (17). In brief, we performed rigorous disinfection for the penis and a constant temperature (37°C) semen collecting cup was used to obtain the samples. Then, the semen samples were filtered by three layers of gauze to remove the gelatinous protein. Immediately after filtration, the concentrated semen samples were sent back to the laboratory. After quality control, the sperm samples with total motility over 80% and density of more than 2 × 10^8^ sperm/ml were retained. To avoid the effect of individual differences on experimental outcomes, 60 mL of qualified semen from each boar was mixed as one sample. The Beltsville thawing solution (BTS) was prepared by dissolving 37 g Glucose, 1.25 g EDTA-2 Na, 6 g Sodium citrate, and 0.75 g Potassium chloride, 1.25 g Sodium bicarbonate,0.6 g penicillin, and 1 g streptomycin sulfate in 1L distilled water. The BTS was supplemented with different concentrations of CAPE as the experimental extenders. The semen sample was diluted to 3 × 10^7^ sperm/ml in each experimental extender.

### Experimental Design

In this study, two experiments were performed.

#### Experiment I

Spermatozoa were incubated in BTS addition of 0, 70, 140, 210, 280, or 350 μmol/L CAPE at room temperature and then stored at 17°C. Each treatment has five repetitions. To determine the optimal working concentration, sperm motion parameters (total motility and progressive motility) and functional integrity (mitochondrial activity, integrity of acrosome, and plasma membrane) were regularly observed from day 1 to 5.

#### Experiment II

To further demonstrate the protective mechanism of CAPE in boar semen liquid preservation, 400 μM H_2_O_2_ was used to construct an oxidative stress model and the optimum concentration of CAPE was applied to evaluate the protective effects. Experimental groups are as follows: the control group; the CAPE group; the H_2_O_2_ group; the co-incubation of CAPE and H_2_O_2_ group. Each group consisted of five replicates. Sperm total and progressive motility, mitochondrial activity, and integrity of acrosome, and plasma membrane on days 1, 3, 5, 7, and 9 were measured to assess sperm quality. Sperm antioxidant capacity was investigated by measuring SOD, CAT, and GSH-Px activity on days 1, 3, 5, and 7. After 9 days of preservation, the expressions of marker proteins AMPK, phospho-AMPKα (p-AMPK), SOD1, and Caspase-3 were analyzed by Western blot and a p-AMPK immunofluorescence assay was performed as well.

### Sperm Motion Parameter

The procedure of sperm motion parameter measurement including total motility and progressive motility by using a Computer-aided sperm analysis system (CASA) (Hamilton IVOS V3, USA) was previously reported in our study (17). Briefly, a 2 ml diluted semen sample (1 × 10^6^ sperm/ml) was pipetted on pre-heated disposable chamber slides (Leja Sperm Counting Slide, Netherlands) at 37°C. Five fields totally contained at least 200 spermatozoa were randomly selected for observation to make the results more accurate. The parameters of CASA were set up according to the manufacturer's instructions. Total motility and progressive motility were automatically calculated by CASA.

### Plasma Membrane Integrity, Acrosomal Integrity, and Mitochondrial Activity

The LIVE/DEAD Sperm Viability Kit (Thermo Fisher Scientific, USA) was used to evaluate plasma membrane integrity. At first, semen samples were incubated with 20 μL SYBR-14 (100 μL of a 1 mM solution in DMSO) for 5 min at 37°C in a dark environment. Then, 20 μL PI (5 mL of a 2.4 mM solution in water) was added for subsequently 5 min incubation. As shown in Supplementary Figure S1A, three types of sperm were observed: (I) sperm with complete plasma membrane was stained with bright green fluorescence (SYBR-14^+^/PI^−^), (II) sperm with damaged plasma membrane exhibited dyed bright red fluorescence (SYBR-14^−^/PI^+^), (III) sperm stained with both green and red fluorescence (SYBR-14^+^/PI^+^) were regarded as owning damaged plasma membrane as well. According to the protocol reported by Fazeli et al. (18), the modified fluorescence staining assay was utilized to assess the morphology of sperm acrosome. In brief, 100 μL 3% polyvinyl pyrrolidone solution was mixed with an equal volume of semen. After centrifugation, the precipitate was diluted by phosphate-buffered saline (PBS) solution to an appropriate concentration. 100 μL diluted solution was used for slide smear and the slide was dried in the air. Subsequently, the mixture of 4',6-diamidino-2-phenylindole (DAPI) (5 μg/ml) and fluorescein isothiocyanate (FITC-PNA) (1.2 μg/mL) was pipetted on the slide and incubated at 37°C. Green fluorescence evenly distributed on spermatozoa head was recognized as with intact acrosome and unevenly distribution as the damaged (PNA-FITC^+^/DAPI^+^, Supplementary Figure S1B). Mitochondrial activity was detected by the 2-(6-Amino-3-imino-3H-xanthen-9-yl) benzoic acid methyl ester (RH123) and propidium iodide (PI) fluorescein staining (19). Briefly, 100 μL hydroxyethyl piperazineethanesulfonic acid buffer (HEPES), 50 μL semen sample, 1 μL RH123 (5 μg/mL), and 1 μL PI (5 μg/mL) solution was mixed and transferred to 37°C water bath. After 15 min incubation, 20 μL solution was used for preparing of slide smear. The air-dried slide was observed by fluorescence microscope (TE2000, Nikon, Japan) and sperm with the green or red fluorescence-stained tail was regarded as possessing or losing mitochondrial activity (Supplementary Figure S1C).

### Determination of SOD, CAT, and GSH-Px Level

To detect sperm antioxidant capacity, total protein was firstly extracted by Triton X-100 (1%) incubation at room temperature for 5 min and centrifugation at 15,000 × g at 4°C for 15 min. The concentration of total protein was determined by a total protein assay kit (BCA quantitative method, Nanjing Jiancheng Bioengineering Institute, Jiangsu, China, A045-3-2). Subsequently, Sperm glutathione peroxidase (GSH-Px), superoxide dismutase (SOD), and catalase (CAT) activities were measured by corresponding assay kits (GSH-Px: A005-1-2, SOD: A001-1-2, and CAT: A007-1-1) according to the manufacturer's (Nanjing Jiancheng Bioengineering Institute) illustrations. Five replicates were performed for each assay. SOD, CAT, and GSH-Px activity of each sampleweres converted into units per mg of total protein in spermatozoa.

### Western Blotting

To obtain enough sperm protein for Western blot analysis, sperm samples were firstly broken by using an ultrasonic disintegrator (setting parameter: three cycles 60% amplitude, 1 s 30 burst, and one time 40% amplitude, 1 s 30 bursts) and then lysed by using 1% Sodium dodecyl sulfate (SDS) solution containing 1 mmol/L Phenylmethylsulfonyl fluoride (PMSF) on ice for 20 min. The samples were centrifuged at 13,600 × g for 2 min at 4°C to collect the supernatant. A proportion of the supernatant was used for total protein quantity analysis and the rest were mixed with 6 × protein loading buffer heated to 95°C for 8 min. The lysates containing equal amounts of protein (20 μg) were loaded on 8%, 10%, 12%, 15% SDS-polyacrylamide gel electrophoresis (SDS-PAGE) to separate AMPK and p-AMPK, GAPDH, Caspase-3, and SOD1, respectively. The target proteins were transferred to the polyvinylidene fluoride (PVDF) membrane, which was blocked with a 5% non-fat milk buffer. Tris-buffered saline containing 1% Tween-20 (TBST) was used to wash the membranes three times. Then, PVDF membranes were incubated with anti-AMPK (1:500, Proteintech, China, Catalog: 10929-2-AP), anti-p-AMPK (1:500, Affinity, America, Catalog: AF3423), anti-Caspase-3 (1:500, ABclonal, China, Catalog: A2156), anti-SOD1 (1:500, ABclonal, China, Catalog: A0274), anti-GAPDH (1:8000, ABclonal, China, Catalog: AC001) at 4 °C overnight. After washing three times, the membranes were incubated with the secondary antibody (HRP Goat Anti-Rabbit IgG (H+L), 1:10000, ABclonal, China, Catalog: AS014) for 2 h at room temperature. The enhanced chemiluminescent (ECL) kit (New Cell & Molecular Biotech, SuZhou, China) was used for protein imprinting detection and the results were analyzed by using Image lab software (Bio-Rad, USA).

### Immunocytochemistry

After washing with PBS solution, sperm samples were fixed with 4% paraformaldehyde for 15 min. Immediately, sperm samples were permeabilized with 0.3% Triton X-100 for 20 min and washed with PBS three times. To prevent non-specific binding, 5% BSA was used to block for 1 h at room temperature. After that, sperm samples were incubated with the first antibody (Rabbit anti-Phospho-AMPKα (Thr172), Santa Cruz Biotechnology, 1:100) overnight at 4°C. The samples were washed by PBS three times and re-suspended with secondary antibody (FITC conjugated Goat Anti-Rabbit IgG H+L from ABclonal, 1:200) for 1 h at 37°C. After washing with PBS three times, the sperm nucleus was stained with DAPI (5 μg/ml) (Solarbio Institute of Biotechnology, 1:1000) for 5 min. For each sample, image scanning was performed in five randomly selected areas containing at least 200 sperm. The fluorescence intensity was determined by five replicates' measurements.

### Statistical Analysis

The experimental data were processed by SPSS software (version 22, SPSS Inc., Chicago, USA). The normality and homogeneity of data were analyzed by the Shapiro-Wilk test and Levene test, respectively. One-way ANOVA (with repeated measures) with the Tukey test was used to compare sperm total motility, progressive motility, plasma membrane integrity, mitochondrial activity, acrosomic integrity, antioxidant capacity, and marker protein expressions in different experimental groups. *P* < 0.05 was set as a statistically significant threshold.

## Results

### Experiment I

#### Effects of Different Concentrations of CAPE on Sperm Quality Parameters

**Figure 1:**
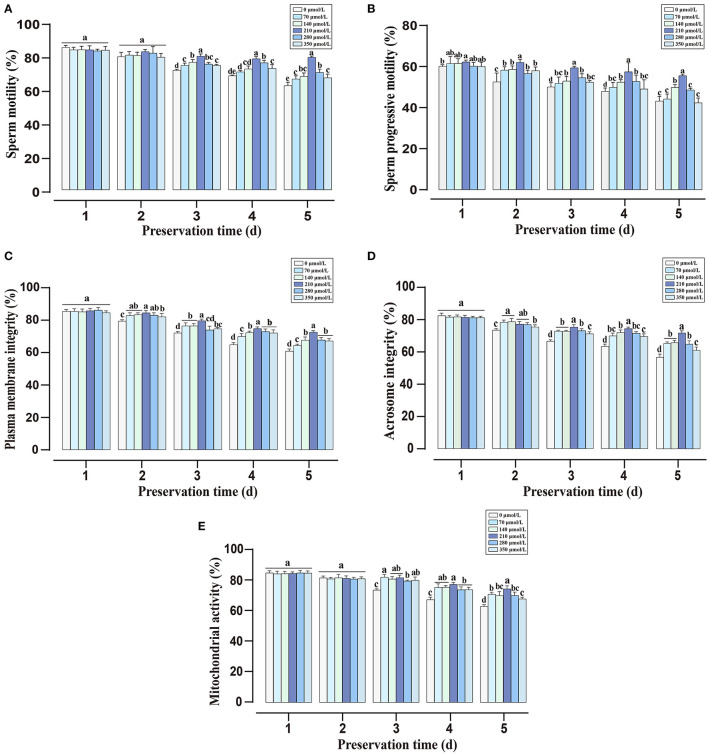
Effects of different concentrations of CAPE on sperm quality parameters during liquid preservation. **(A)** Sperm total motility. **(B)** Sperm progressive motility. **(C)** Plasma membrane integrity. **(D)** Acrosome integrity. **(E)** Mitochondrial activity. Results are presented as mean ± SD (*n* = 5). Different letters (a–e) represent for significant differences (*P* < 0.05).

During liquid storage, the alternations of sperm total and progressive motility are shown in Figures 1A,B. There were no significant differences in total motility among all groups at the initial time (day 1 and day 2). With the extension of preservation time, supplementation with CAPE significantly improved the total motility compared with the control group (*P* < 0.05), and the 210 μmol/L CAPE treatment group possessed the highest value. Similarly, compared to the control group, the addition of CAPE distinctly increased the progressive motility, and supplementation with 210 μmol/L CAPE was the most effective. As for sperm plasma membrane integrity, mitochondrial activity, and acrosomic integrity, the index values of CAPE supplementation groups were significantly higher than those of the control group with prolonging the preservation time (Figures 1C–E, *P* < 0.05). At the end of preservation, although the index values in all group declined, the 210 μmol/L CAPE group exhibited minimal reductions. Due to the addition of 210 μmol/L CAPE in the extender exerted the excellent and integrated protective roles in sperm liquid storage, the concentration was used in the further experiments.

### Experiment II

#### Effect of CAPE in the Presence of H_2_O_2_ on Sperm Quality Parameters

**Figure 2:**
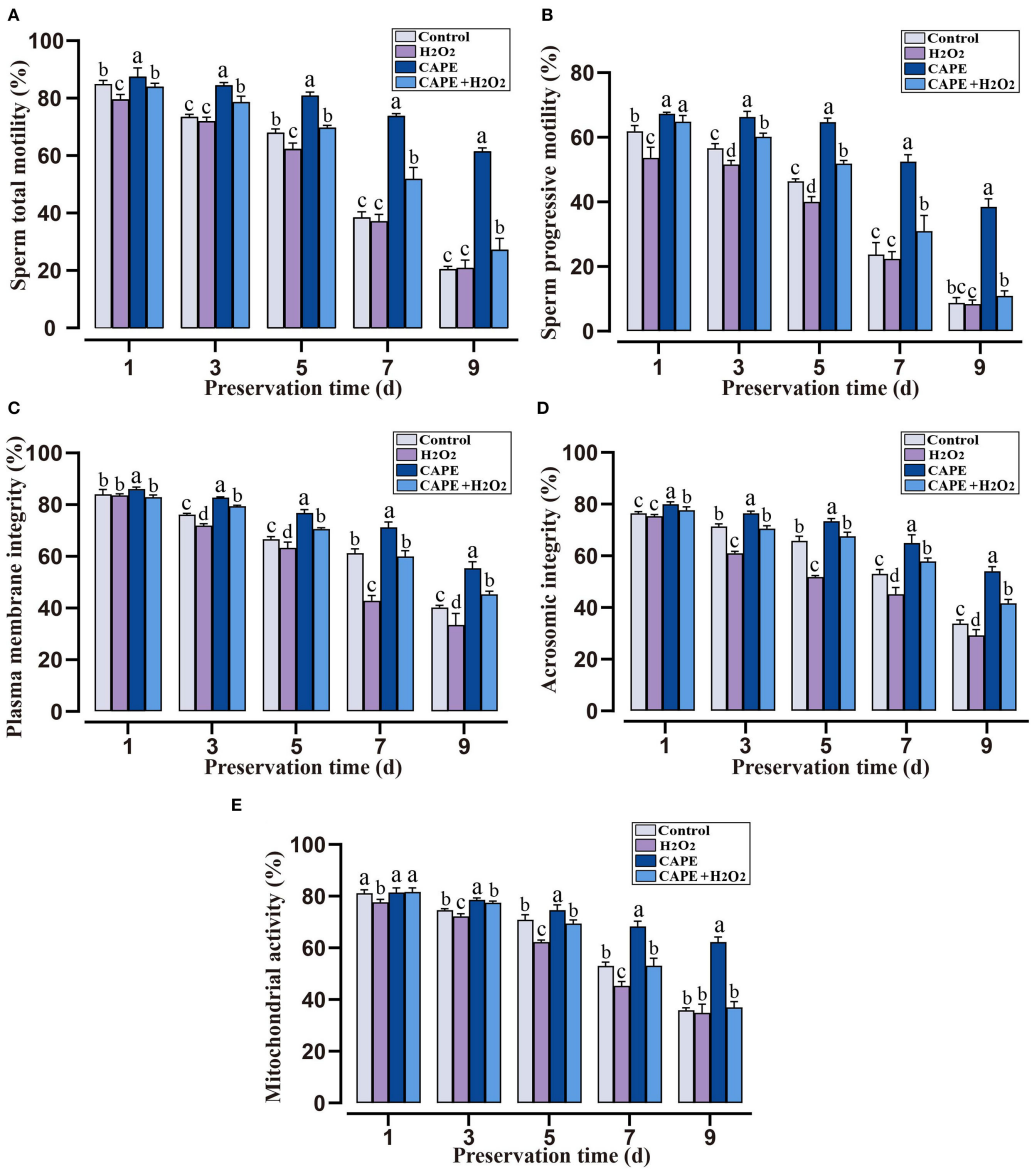
The protective roles of CAPE in sperm motion and functional integrity parameters in oxidative stress or normal state. **(A)** Sperm total motility; **(B)** Sperm progressive motility; **(C)** Plasma membrane integrity; **(D)** Acrosomic integrity; **(E)** Mitochondrial activity. Mean ± SD was used for the exhibition of the results (*n* = 5). Significant differences (*P* < 0.05) were uncovered by different letters (a–d).

To investigate the role of CAPE in alleviating H_2_O_2_-induced sperm quality decay, sperm quality parameters under different treatments were observed. Within 9 days of liquid storage, both the total motility and progressive motility remained significantly higher (*P* < 0.05) in CAPE treated groups compared to untreated groups even in the presence of H_2_O_2_ (Figures 2A,B). Although the plasma membrane integrity, acrosomic integrity, and mitochondrial activity gradually decreased over the preservation time, these index values declined more rapidly in groups without CAPE treatment (presences or absences of H_2_O_2_, Figures 2C–E, *P* < 0.05).

#### Effect of CAPE in the Presence of H_2_O_2_ on Sperm Antioxidant Capacity

**Figure 3:**
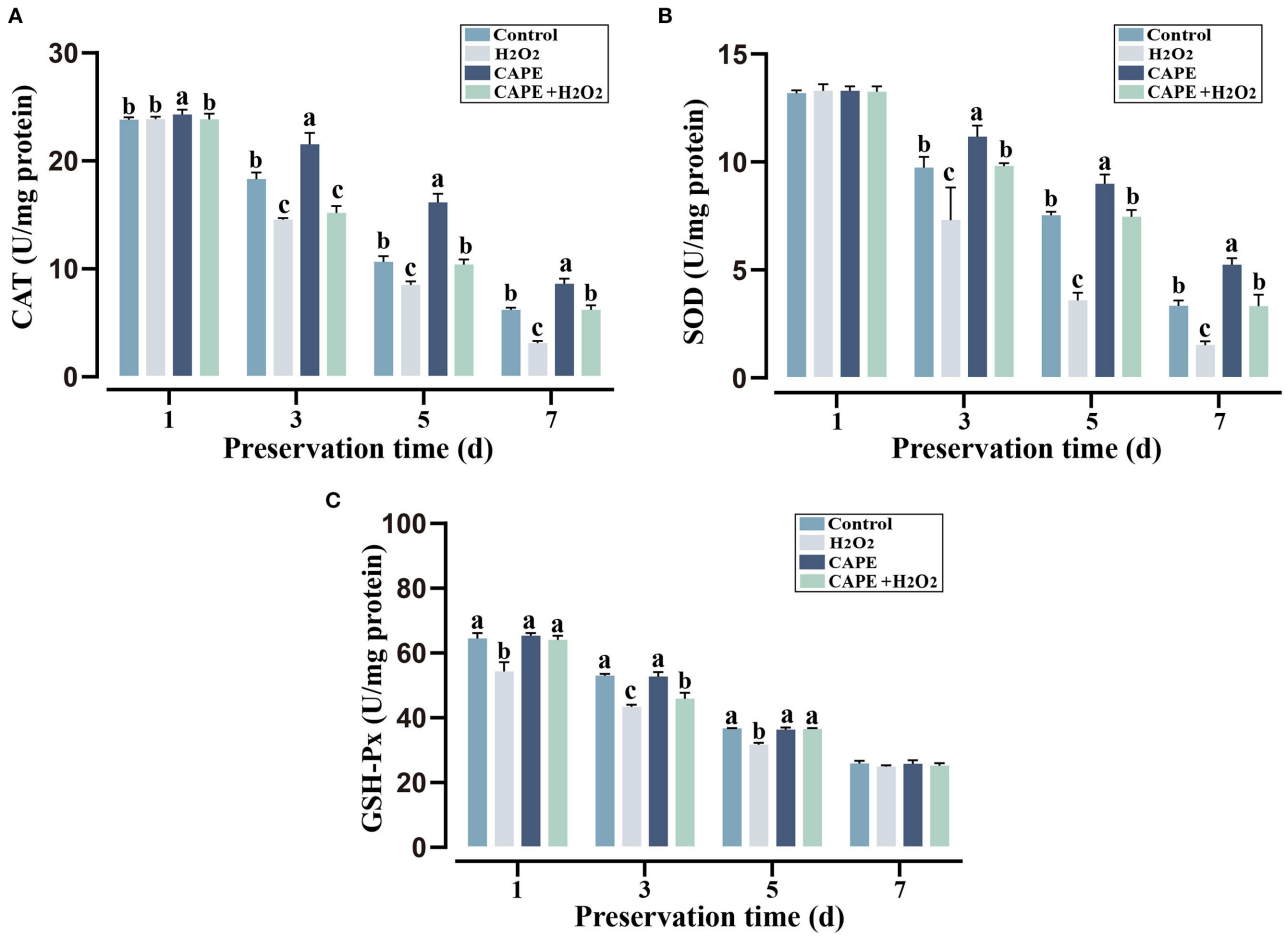
CAPE affected sperm antioxidant capacity among treatment and control groups. **(A)** CAT activity; **(B)** SOD activity; **(C)** GSH-Px activity. Results are expressed as mean ± SD (*n* = 5). Different letters (a–c) indicated significant differences (*P* < 0.05).

To determine the protective effect of CAPE against H_2_O_2_ triggered oxidative stress, sperm antioxidant capacity in different preservation extenders was measured. During the preservation process, the CAT, SOD, and GSH-Px activities declined with the preservation time in various groups (Figure 3). On the seventh day of storage, sperm after CAPE treatment had greater CAT and SOD activities in the presence or absence of H_2_O_2_ (*P* < 0.05), but the GSH-Px activity was not distinctive.

#### Sperm AMPK, p-AMPK, SOD1, and Caspase-3 Activity

**Figure 4:**
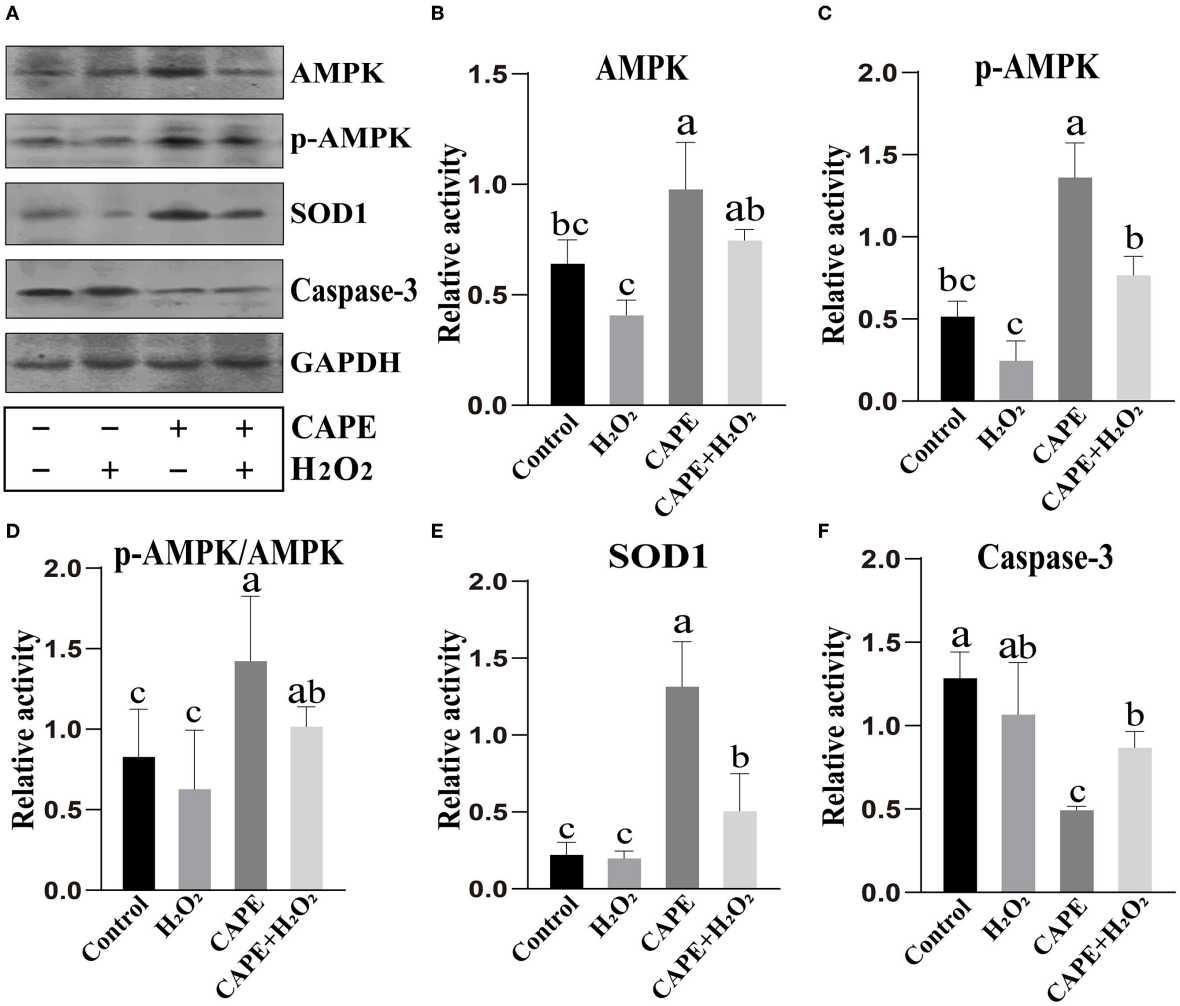
Western blot analysis. **(A)** The protein activity of AMPK, p-AMPK, SOD1, Caspase-3, and GAPDH; **(B)** Quantitative analysis of AMPK; **(C)** Quantitative analysis of p-AMPK. **(D)** The proportion of p-AMPK to AMPK; **(E)** Quantitative analysis of SOD1; **(F)** Quantitative analysis of Caspase-3. Graph bars represent mean ± SD (*n* = 3). Different letters (a–c) showed significant differences (*P* < 0.05).

**Figure 5:**
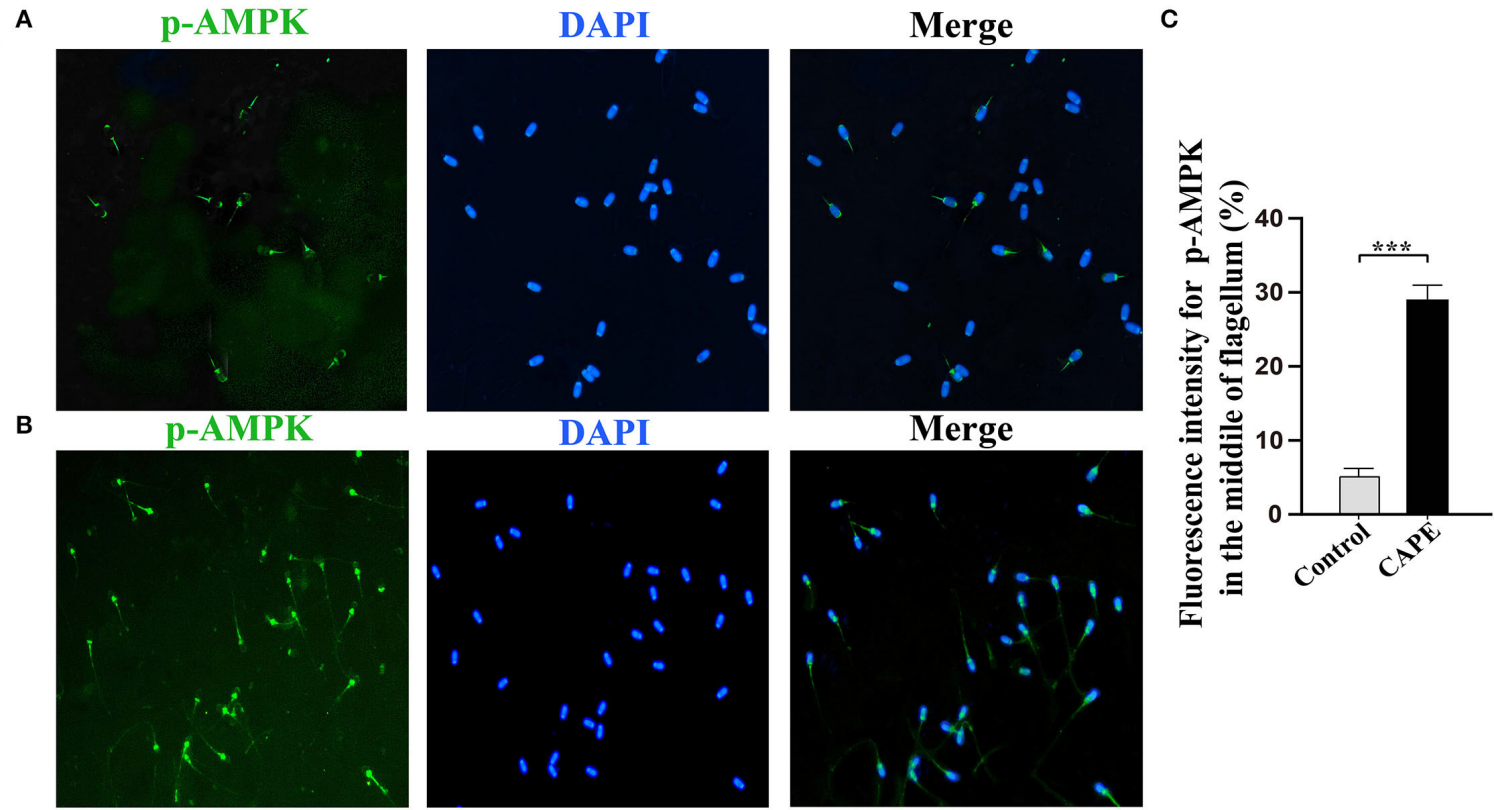
Subcellular localization of p-AMPK by Immunocytochemistry. **(A)** The signal was found in the acrosome and midpiece of the flagellum in the control group; **(B)** The signal is present at the midpiece of the flagellum in the CAPE treatment group; **(C)** The relative fluorescence intensity of p-AMPK in the control and CAPE treatment groups (mean ± SD, *n* = 3, asterisks consider as significant differences, *P* < 0.001).

To understand the underlying mechanism by which CAPE exerts protective effects on sperm, the activity of AMPK, p-AMPK, SOD1, and Caspase-3 at the end of preservation was evaluated by Western blot. As shown in Figure 4, CAPE significantly alleviated the protein degradation of AMPK, p-AMPK, and SOD1 and depressed Caspase-3 activity regardless of whether H_2_O_2_ presence or not (*P* < 0.05). To further solid our evidence, an immunofluorescence assay was performed to assess the p-AMPK level between CAPE-treated and control groups. In line with the western blot data, the result showed that the CAPE-treated group exhibited more positive signals of p-AMPK than the control group (Figure 5).

## Discussion

Liquid preservation of sperm is a common procedure for preserving male fertility in human and domestic animals, but this process is accompanied by oxidative stress and excessive free radical (ROS) production with the prolonging of preservation time. Accumulating evidence demonstrated that the activation of AMPK inhibits ROS production which implies its potential application in sperm preservation (20–22). Although CAPE could activate AMPK (15, 16), whether it has a protective effect on boar sperm has not been determined. In this study, we assess the effects of CAPE on different parameters of boar sperm during liquid preservation at 17°C and choose the appropriate CAPE concentration to investigate its protective mechanism. Our findings suggested that 210 μmol/L CAPE supplementation in the extender distinctly improved boar sperm quality and antioxidant capacity. The protective role was linked to the activation of AMPK activity.

Caffeic acid phenethyl ester is a natural bioactive compound of propolis extract that possesses antioxidant property, which has been regarded as a potential inhibitory agent of infertility (23). Here, we found that the addition of CAPE to the extender significantly improved sperm motility, plasma membrane integrity, and acrosomal integrity (Figure 1). Especially, sperm treated with 210 μmol/L CAPE exhibited significantly higher quality parameters than those in the control group and other treatment groups even in the presence of H_2_O_2_ (Figures 1, 2). Wang et al. demonstrated that CAPE is a polyphenol with hydroxyl groups within the catechol ring which is responsible for its cytoprotective role (24). In addition, Burgazli et al. indicated that CAPE modulated intracellular Ca^2+^ homeostasis in human endothelial cells in a concentration-dependent manner which affects cellular integrity and function, but an increasing concentration of CAPE can result in damaging effects (25). This result could be used to explain the higher concentration of CAPE causes a reduction in sperm quality. It is generally believed that H_2_O_2_ is one of the most toxic forms of ROS in semen (26). Consequently, boar sperm incubated with H_2_O_2_ induced a rapid decrease in antioxidant enzymes (CAT, SOD, and GSH-Px) activities (Figure 3). In contrast, supplementation with CAPE could attenuate this process. Although the antioxidant effects of CAPE have been extensively studied in liver, colon, and kidney cells (27–29), only a few studies indicated that the antioxidant activity of CAPE can protect sperm from oxidative damage (10, 30).

Adenosine monophosphate-activated protein kinase plays a crucial role in the maintenance of cellular energy homeostasis, which contributes to modulating sperm motility, mitochondrial activity, plasma membrane integrity, and acrosomic integrity (31). AMPK could be activated by several physiological factors, hormones, and drugs including oxygen or glucose starvation, adiponectin, and metformin (32, 33). Recently, CAPE as a natural product that activates and regulates AMPK activity has attracted widespread interest. For instance, Lee et al. disclosed that CAPE-activated AMPK could stimulate glucose uptake in skeletal muscle cells (34). Tsai et al. revealed that CAPE mediated anti-neuroinflammatory responses in microglial cells through AMPK activation (35). Although there is no available evidence indicating that AMPK could be activated by CAPE in sperm cells, the regulatory roles of CAPE in several signaling pathways of protein degradation such as inhibitor kappaB and HIF-1alpha protein may be implicated in AMPK activity maintenance (36, 37). Consequently, the depletion of both AMPK and the activation form of AMPK (p-AMPK) was effectively prevented by the CAPE treatment observed in the present study (Figure 4). In addition, we also found that the activity of indicator protein of antioxidant status (SOD1) and apoptosis (Caspase-3) induced by oxidative stress was conserved and depressed by CAPE incubation, respectively. This result was in accordance with the findings of previous research on the role of CAPE in antioxidant defense regulation (38, 39). Despite our data showing that CAPE could maintain AMPK activity which induced the improved sperm quality and antioxidant capacity even with H_2_O_2_ co-incubation, the underlying mechanism by which AMPK is involved in CAPE-mediated protective effects was not unveiled in the present study. Nonetheless, the gene expression of peroxisome-proliferator-activated receptor gamma coactivator 1alpha (PGC-1α) induced by AMPK which further activated nuclear factor erythroid 2-related factor 2 (Nrf2)-hemeoxygenase-1 (HO-1) axis is essential for the antioxidant properties of CAPE should be one of the possible mechanism (28, 40, 41).

## Conclusions

In the current study, we have determined that the basic extender supplementation with 210 μmol/L CAPE could effectively alleviate boar sperm quality decay and oxidative stress status during liquid storage at 17°C in the presence or absence of H_2_O_2_. Future studies should focus on obtaining a comprehensive and deep insight into the molecular mechanism of CAPE exerts protective effects on boar sperm related to AMPK activity maintenance.

## Data Availability Statement

The raw data supporting the conclusions of this article will be made available by the authors, without undue reservation.

## Ethics Statement

The animal study was reviewed and approved by Animal Care and Use Committee (ACUC) in Fujian Agriculture and Forestry University.

## Author Contributions

TX conceived and designed the experiments, supervised the experiment progress, and revised the manuscript. SF designed the experiments, analyzed the data, and wrote and revised the manuscript. QL performed the experiments, analyzed the data, and wrote the manuscript. LX, JC, and YX performed the experiments. All authors read and approved the final manuscript.

## Funding

This work was supported by grants from the Natural Science Foundation of Fujian Province (2020J01537) and the Modern Agricultural Technology System Program of Fujian Province (2019-144).

## Conflict of Interest

The authors declare that the research was conducted in the absence of any commercial or financial relationships that could be construed as a potential conflict of interest.

## Publisher's Note

All claims expressed in this article are solely those of the authors and do not necessarily represent those of their affiliated organizations, or those of the publisher, the editors and the reviewers. Any product that may be evaluated in this article, or claim that may be made by its manufacturer, is not guaranteed or endorsed by the publisher.

## Supplementary Material

Supplementary Figure S1 Fluorescence detection of boar sperm functional integrity. **(A)** Distributions of fluorescent on sperm plasma membrane integrity; I, Sperm with intact plasma membrane; II and III, Sperm with damaged plasma membrane; **(B)** Distributions of fluorescent on acrosome integrity; I, Sperm with damaged acrosome; II, Sperm with intact acrosome; **(C)** Distributions of fluorescent on mitochondrial activity. I, Sperm losses mitochondrial activity; II, Sperm possesses mitochondrial activity.

Supplementary Table S1 The comparison analysis between control and 210 μmol/L CAPE treatment group of phospho-AMPKα antibody labeling in sperm tail (^***^, *P* < 0.001).
