# Supplementary figures and images for: Caffeic Acid Phenethyl Ester (CAPE) Improves Boar Sperm Quality and Antioxidant Capacity in Liquid Preservation (17°C) Linked to AMPK Activity Maintenance
Source: Front Vet Sci. 2022 Jun 9;9:904886. doi: 10.3389/fvets.2022.904886 (PMC9219730; doi:10.3389/fvets.2022.904886)

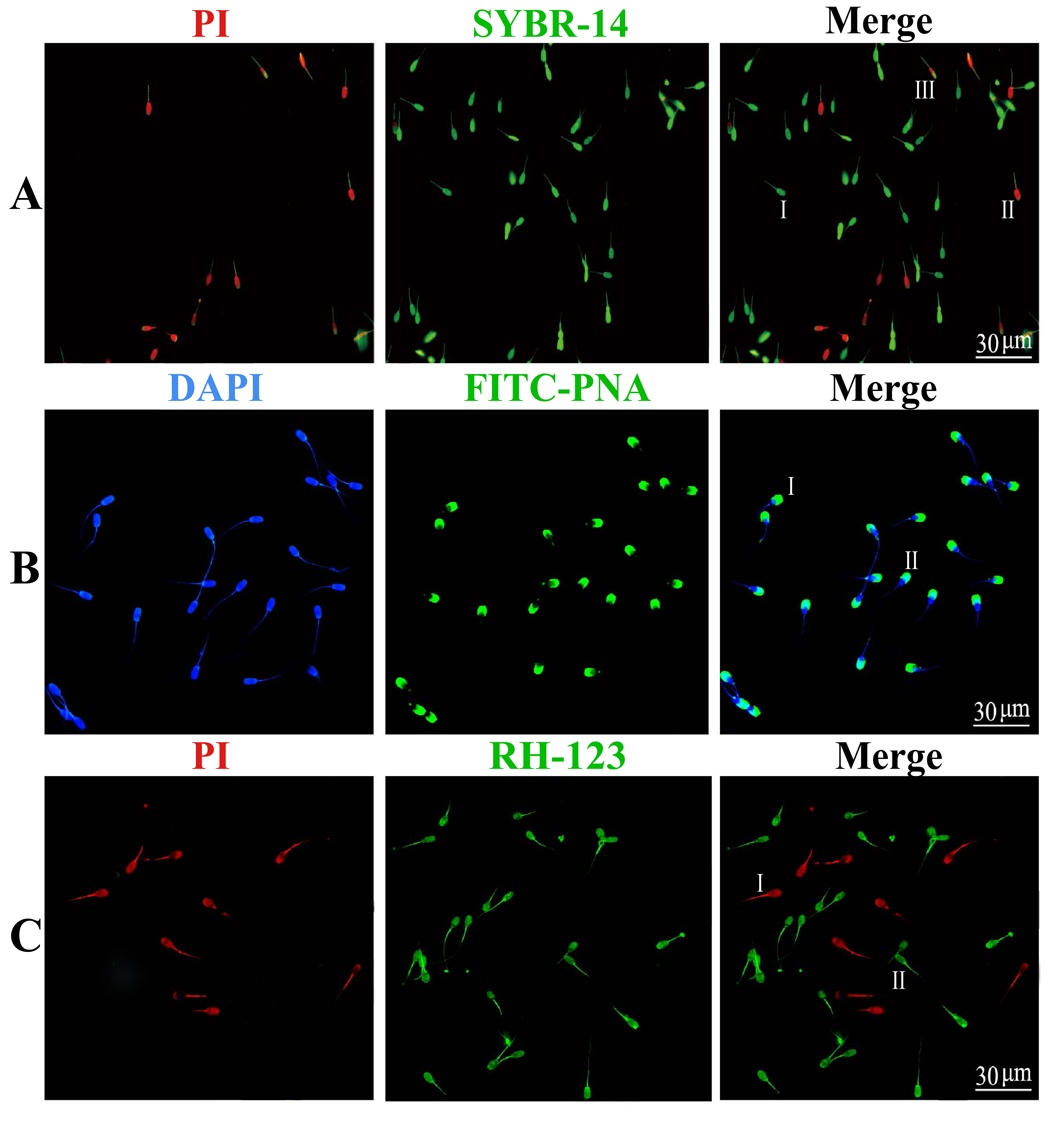

Supplement: Supplementary Figure S1 — Fluorescence detection of boar sperm functional integrity. (A) Distributions of fluorescent on sperm plasma membrane integrity; I, Sperm with intact plasma membrane; II and III, Sperm with damaged plasma membrane; (B) Distributions of fluorescent on acrosome integrity; I, Sperm with damaged acrosome; II, Sperm with intact acrosome; (C) Distributions of fluorescent on mitochondrial activity. I, Sperm losses mitochondrial activity; II, Sperm possesses mitochondrial activity. [file Image_1.JPEG]

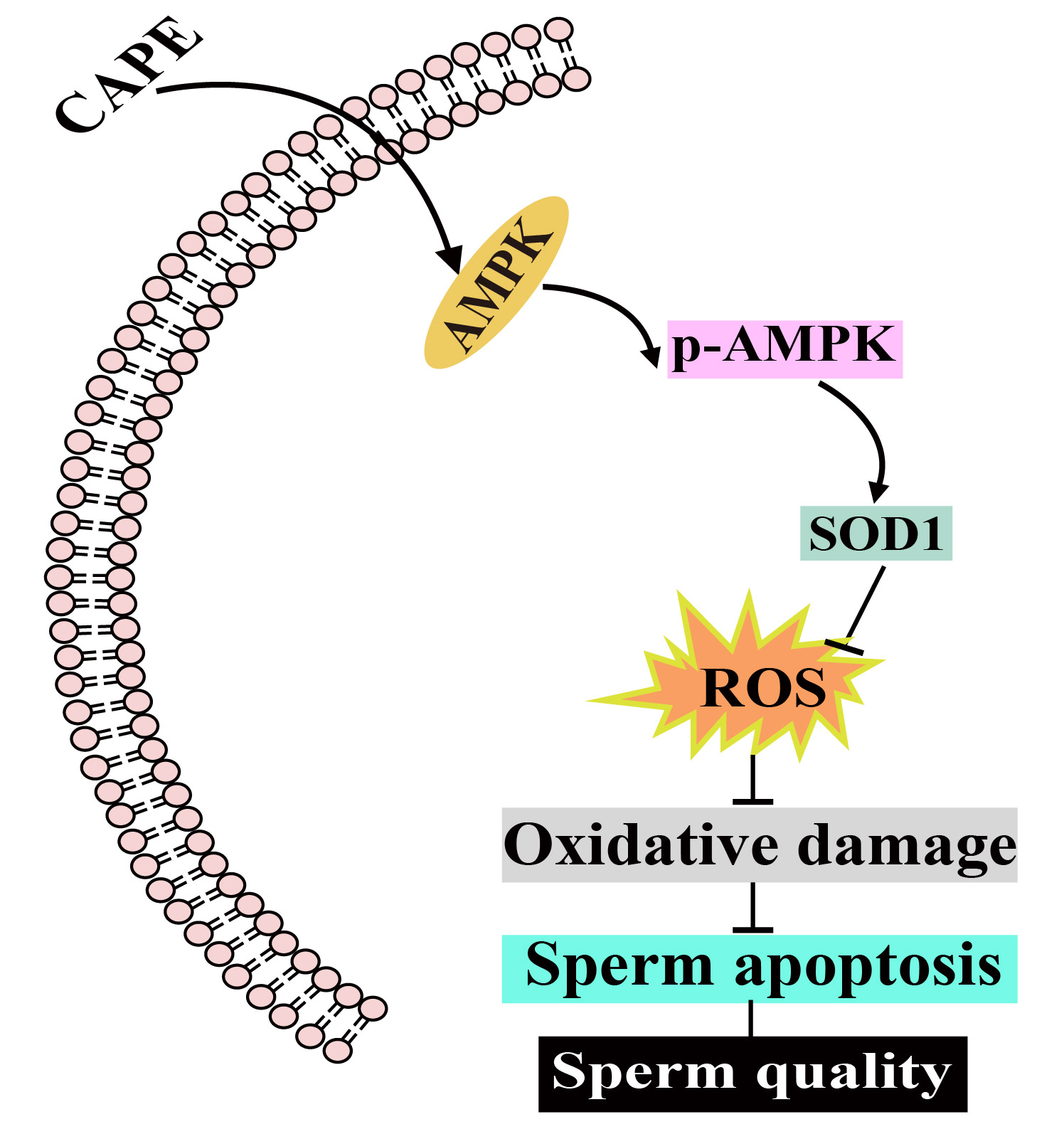

Supplement: Supplementary file 3 [file Image_2.JPEG]
